# Interaction between *Mycobacterium tuberculosis*, *Mycobacterium bovis*, *Mycobacterium avium *subspecies *paratuberculosis *with the enteric glia and microglial cells

**DOI:** 10.1186/1757-4749-3-19

**Published:** 2011-12-09

**Authors:** Sara Cannas, Paola Molicotti, Alessandra Bua, Donatella Usai, Leonardo A Sechi, Antonio M Scanu, Elisabetta Blasi, Stefania Zanetti

**Affiliations:** 1 Dipartimento di Scienze Biomediche - Microbiologia Sperimentale e Clinica. Università degli Studi di Sassari, Italy; 2 Dipartimento di Medicina Clinica Sperimentale ed Oncologica - Sezione Clinica Chirurgica. Università degli Studi di Sassari, Italy; 3 Dipartimento di Scienze di Sanità Pubblica, Università di Modena e Reggio Emilia, Italy

**Keywords:** Mycobacteria, enteric glial cells, microglia, cytokines

## Abstract

**Background:**

We investigated the interaction of *Mycobacterium avium *subspecies *paratuberculosis, M. bovis *and *M. tuberculosis *and different glial cells (enteric glial and microglial cells) in order to evaluate the infecting ability of these microorganisms and the effects produced on these cells, such as the evaluation of cytokines expression.

**Results:**

Our experiments demonstrated the adhesion of *M. paratuberculosis *to the enteroglial cells and the induction of IL-1A and IL-6 expression; *M. tuberculosis *and *M. bovis *showed a good adhesive capability to the enteric cell line with the expression of the following cytokines: IL-1A and IL-1B, TNF-α, G-CSF and GM-CSF; *M. bovis *induced the expression of IL-6 too.

The experiment performed with the microglial cells confirmed the results obtained with the enteroglial cells after the infection with *M. tuberculosis *and *M. bovis*, whereas *M. paratuberculosis *stimulated the production of IL-1A and IL-1B.

**Conclusion:**

Enteroglial and microglial cells, could be the target of pathogenic mycobacteria and, even if present in different locations (Enteric Nervous System and Central Nervous System), show to have similar mechanism of immunomodulation.

## Background

Together with neurons, glial cells form the nervous system; they have nutritive and supportive function also for neurons, isolate the nervous tissues and protect them from foreign bodies in case of injuries.

These cells are known as microglial cells in the Central Nervous System (CNS), and as enteric glial cells in the Enteric Nervous System (ENS); they have identical morphologic and functional features and are functionally and immunologically correlated to the monocyte/macrophage cells.

In Irritable Bowel Syndrome (IBS) and Inflammatory Bowel Disease (IBD), especially in Crohn's disease, a neurodegenerative state is steady. Therefore, changes in the nervous functions could represent an important link between inflammation and neurodegeneration, and this link could be represented by the glial cells, which have demonstrated to control the enteric neuronal functions [1-5]. In particular, alterations of the glial cells may be responsible of the increase of the mucosal barrier permeability, of the neuronal cells' proliferation and of the production of neurokines.

All this confirms the leading role performed by the enteric glia in the inflammation and therefore it could be regarded as an important source of cytokines in the neuroimmune network of the intestine [1-5].

CNS tuberculosis and brain tuberculoma are one of the most serious manifestations of tuberculosis, accounting for 1%-10% of all cases [6,7]. The infection with MTB leads to an inflammatory tissue destruction [8]. The mechanisms behind this phenomenon are nowadays unknown. The pathogenesis, diagnosis and treatment of CNS-TB have received little attention. A better understanding of its pathogenesis is important to improve existing therapies.

CNS-resident macrophages and microglia are infected with MTB, these cells may be the main cellular target in the CNS [9,10]. A peculiar characteristic of this bacillus is its ability to infect and multiply inside these cells [6,9]. Microglial cells reside within the nervous system's parenchyma and in their inactive or resting state have a characteristic "branched" morphology, never seen in resident macrophages of other systems. Microglial cells, in response to a variety of insults such as infection, traumatic injury or ischemia, assume an ameboid shape, and move towards the site of injury [7,11]. Microglia showing its ameboid phenotype increases its proliferation [12], motility [13], fagocytic activity [14,15] and release of cytokines and reactive oxygen [16-18]. Activated macrophages are key elements in the antimycobacterial immunity: in fact, they secrete specific cytokines against these microorganisms. In particular, the tumor necrosis factor-α (TNF-α) with type 1 cytokines (IFN-γ and IL-1) are essential in the immune response and could be important factor in the immune pathology [6,19,20]. Some works have shown that the ingestion of nonopsonized *M. tuberculosis *by the human microglia is facilitated by the receptor CD14 [9]; this receptor, together with the integrin β CD-18 and the TNF-α, is involved in the formation of giant mononucleate cells in the swine microglia infected with *M. bovis *[21].

Moreover, we decided to study the possible interaction between *M. paratuberculosis *and the enteroglial cells, in particular the ability to infect glial cells and their consequent activation in antigen-presenting cells, with relevant production of proinflammatory cytokines. Data obtained were correlated to find out analogies and/or differences in the three pathogenic microorganism towards enteric glial and microglial cells.

## Results

### Adhesion and infection of *M. avium *subsp. *paratuberculosis M. tuberculosis *and *M. bovis *to the enteric glial cells

*M. paratuberculosis, M. tuberculosis *and *M. bovis *showed the ability to adhere to the enteric glial cells after six hours from the infection.

**Figure 1:**
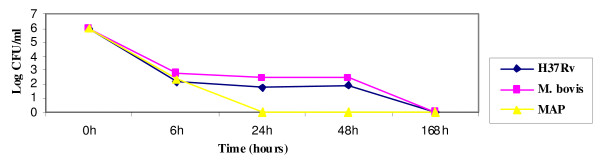
**Growth index of *M. tuberculosis *H37*Rv*, *M. bovis *and *M. paratuberculosis *in enteric glial cells**.

The growth graph pointed out a progressive decrease of CFU/ml within 24 hours for *M. paratuberculosis*, while *M. tuberculosis *and *M. bovis *have the ability to survive the 48 hours. At six hours from the infection, all the strains have the same value of CFU/ml, while already after 24 hours it is possible to observe some differences: in particular *M. paratuberculosis*, although it has an adhesive ability and is vital, does not seem to persist inside the cells, as pointed out by a great decrease of CFU/ml starting from 24 hours from the infection. Regarding *M. tuberculosis *and *M. bovis*, both show the ability to infect and multiply inside the enteric glial cells (Figure 1).

### Expression of cytokines by enteric glial cells after infection with different species of mycobacteria

**Figure 2:**
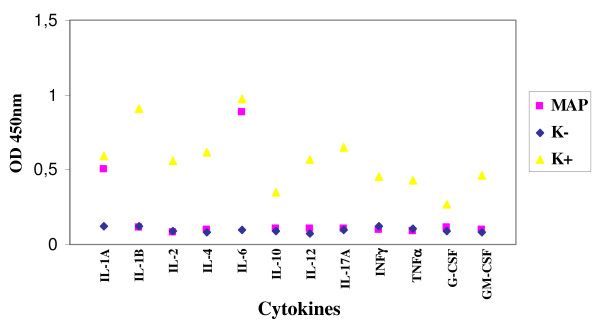
**Expression of cytokines by enteric glial cells MIM/PPK infected by *M. paratuberculosis *1515**.

The experiment of infection realized with the strain *M. paratuberculosis *1515 and enteric glial cells showed a production of IL-1A and IL-6 already after 24 hours after the infection, until the achievement of a plateau after 48 hours (Figure 2). The other cytokines were absent.

**Figure 3:**
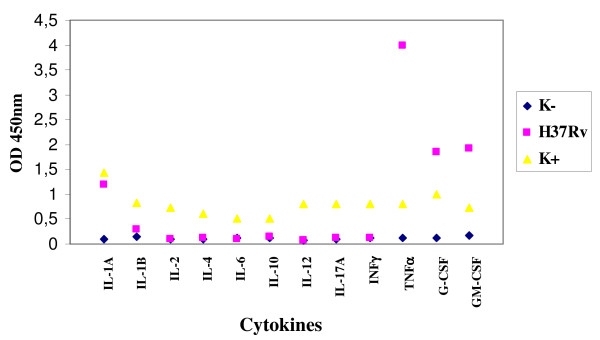
**Expression of cytokines by enteric glial cells MIM/PPK infected by *M. tuberculosis *H37*Rv***.

The infection of enteric glial cells with *M. tuberculosis *H37*Rv *stimulated the production of IL-1A, TNF-α, G-CSF, GM-CSF and, to a lesser extent, of IL-1B (Figure 3).

**Figure 4:**
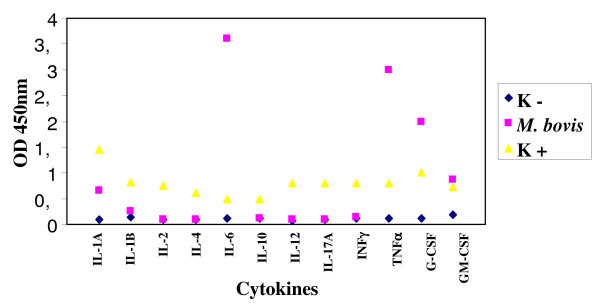
**Expression of cytokines by enteric glial cells MIM/PPK infected by *M. bovis***.

Moreover *M. bovis *stimulated the production besides of IL-1A, IL-1B, TNF-α, G-CSF and GM-CSF, also of IL-6, absent in *M. tuberculosis *and present in a lesser extent in MAP (Figure 4).

### Adhesion and infection of *M. avium *subsp. *paratuberculosis, M. tuberculosis and M. bovis *to microglial cells

**Figure 5:**
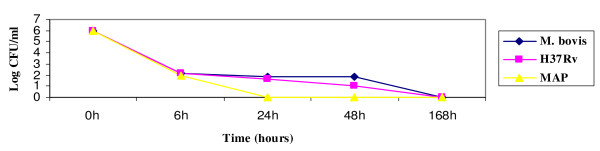
**Growth index of *M. tuberculosis *H37*Rv*, *M. bovis *and *M. paratuberculosis *1515 in microglial cells BV-2**.

The three strains considered show a good ability to adhere to the microglial cells. As pointed out by the growth curve, *M. paratuberculosis *has a progressive decrease of the CFU/ml within 24 hours. The results obtained with the growth curve showed the high capability of survival of *M. tuberculosis *and *M. bovis *after six hours from the infection, and their persistence even after 48 hours, due to their tropism towards these cells. Data obtained have pointed out that *M. tuberculosis *H37*Rv *and *M. bovis *infect and multiply inside the glial cells BV-2, whereas *M. paratuberculosis *does not have the ability to multiply inside these cells (Figure 5)

### Expression of cytokines by microglial cells BV-2 after the infection with different species of mycobacteria

**Figure 6:**
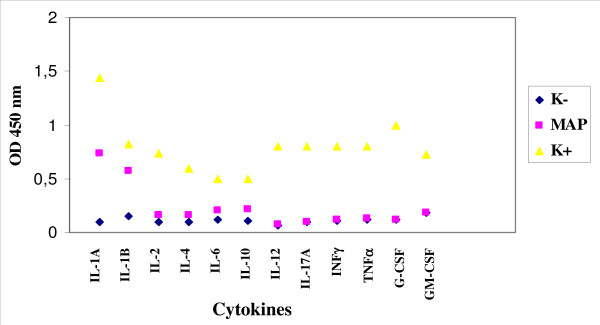
**Expression of cytokines by microglial cells BV-2 infected by *M. paratuberculosis *1515**.

The infection of microglial cells with *M. paratuberculosis *stimulated the expression of IL-1A and IL-1B (Figure 6).

**Figure 7:**
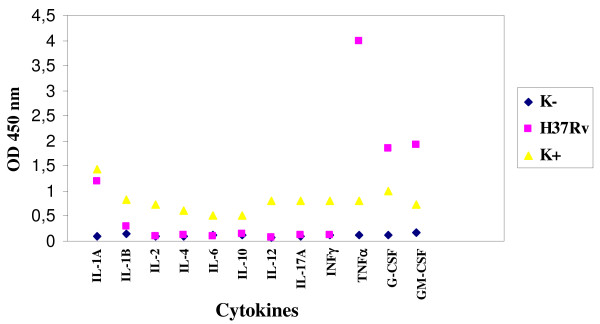
**Expression of cytokines by microglial cells BV-2 infected by *M. tuberculosis *H37*Rv***.

**Figure 8:**
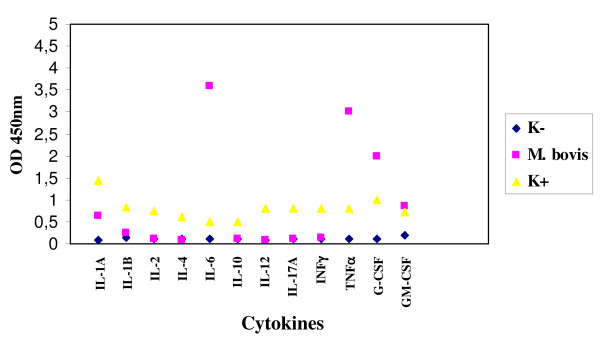
**Expression of cytokines by microglial cells BV-2 infected by *M. bovis***.

Regarding the infection of microglial cells with *M. tuberculosis *and *M. bovis*, these microorganisms stimulated the production of IL-1A, IL-1B, TNF-α, G-CSF and GM-CSF; besides *M. bovis *stimulated the production also of IL-6 (Figures 7 and 8).

## Discussion

The results obtained with the experiments carried out with *M. paratuberculosis*, *M. tuberculosis *and *M. bovis *and enteroglial and microglial cells are overlapping, despite the different anatomical localization of the cell lines tested. The experiments of adhesion and infection "in vitro" with both cell lines showed that *M. paratuberculosis *adhered to these cells already after 6 hours from the infection, but after 24 hours there was a numerical decrease [22].

Previous experiments with this microorganism pointed out that it requires a long time of adaptation inside the infected cells before multiplying (8-10 days between adaptation and replication), a too long time for our cell lines, which already at the fourth day showed signs of stress.

On the contrary, *M. tuberculosis *and *M. bovis *showed a good ability of adhesion and persistence, multiplying inside both the enteroglial and the microglial cells in the 24 hours. Regarding the production of cytokines, both the cell lines, after exposure to *M. tuberculosis *and *M. bovis*, expressed the following: IL-1A and IL-1B, TNFα, G-CSF and GM-CSF. As described in literature, cytokines G-CSF and GM-CSF stimulate the population of macrophages, strengthening the activity of APC through the expression of MHC II. In several experiments of infection of the glial cells it was observed that *M. tuberculosis *causes a suppression of the production of the cytokines related to the virulence of the strain, which is directly proportional to the immunosuppressive ability. These data agree with those obtained in our study, in which we found a scarse production of most cytokines assayed, among which IFN-γ, cytokine involved in the protection towards the mycobacterial infections. Consequently, the scarse expression of cytokines seems to be related to the immunosuppressive effect deriving from the infection with tubercular mycobacteria, both *M. tuberculosis *and *M. bovis*. In a study [6] it was observed that after the infection of microglial cells by *M. tuberculosis*, the production of cytokines IL-1A and IL-10 was prevented. On the contrary, in our work we observed the production of IL-1A and the absence of IL-6, produced instead by *M. bovis *and *M. tuberculosis*. These two cytokines seem to have a functional activity: both can alter the proliferation of the enteric glia [23] that, in its turn, causes a disorder in the mucosal permeability and neuroimmune-modulatory response [23,2]. Many works indicate that in the chronic inflammatory colites there is a high production of IL-1A, as it was pointed out by our results after the infection with *M. paratuberculosis*, consequently it could be hypothesized that there is a strong relation between the presence of this microorganism and these pathologies. Curiously, the role of IL-1A is to recruit leucocytes in the infection site and to take part, together with TNF-α, in the formation and the maintenance of the granuloma. However, despite the high production of this cytokine in the lesions associated to the clinical infection due to *M. paratuberculosis*, there are not leucocytes and neutrophils, and even the production of TNF-α is absent [24]. All this agrees with our results, considering that the expression of TNF-α was not induced by *M. paratuberculosis*, while it was stimulated after the infection of both cell lines by *M. tuberculosis *and *M. bovis*. Considering the important role of TNF-α in the maintenance of the balance host-parasite in the infections caused by these two microorganisms, its absence in the infections due to *M. paratuberculosis *could be related to the chronicity of the pathologies caused by this microorganism.

## Conclusions

In conclusion, our results contribute to clarify the role of microglial and enteroglial cells in the mycobacterial infections. First, enteroglial cells modulate and direct damage to the nervous system through the intestinal immune-mediated mechanism and this explains their constant activation in association with the nerve degeneration. Secondly, cytokine inhibition induced by *M. tuberculosis *and *M. bovis *infection results to immuno-suppression. The final point that emerges from this works is that the results obtained from the experiments conducted on microglial and enteroglial cells are similar despite the different localization of both enteric and central nervous system.

## Methods

### Adhesion and infection of mycobacterial species to enteric glial and microglial cells

We evaluated the adhesive ability of mycobacteria using an "*in vitro*" assay with the murine enteric glial cells MIM/PPk and microglial cells BV-2.

The MIM/PPk murine enteric cell lines were kindly provided by Prof. Anne Ruehl (University of Munich; the BV2 murine microglial cell lines by Prof. Elisabetta Blasi (University of Reggio Emilia and Modena).

The MIM/PPk cells grow in DMEM-F12 (Dulbecco Minimal Essential Medium, GIBCO) with10% of cattle's fetal serum and100 U/ml penicillin and streptomicin. The BV2 cells grow in RPMI 1640 (GIBCO) with 10% of cattle's fetal serum, Gluta MAX, 100 U/ml penicillin, streptomicin and 10 μg/ml of ciprofloxacin. The cells were seeded on slides inside 24-well plates and infected with the mycobacterial strains (1 ml of mycobacteria in log phase), then we tested the expression of cytokines, verified the adhesion and the persistence inside the cells. *M. paratuberculosis 1515 *strain was grown at 37°C in liquid medium 7H9 Middlebrook added with Mycobactine J and Middlebrook ADC. *M. tuberculosis *and *M. bovis *strains were raised at 37°C in liquid medium 7H9 added with Middlebrook ADC. Cell monolayers were washed with PBS and then 1 ml of mycobacteria in log phase was incubated at a MOI (Multiplicity of infection) of 10:1 (approximatively 10^6 ^bacteria for 10^5 ^cells). For each strain the experiment was made in double and repeated three times.

After 6 hours, infected cells were washed with the cell medium containing 100 μg/ml kanamycin and incubated with the same medium. Two non-infected monolayers represented the negative control.

The adhesive ability of the bacteria was pointed out after six hours from the infection. *M. paratuberculosis *was visualized through the auramin/rodamin stain and the slides were observed with the fluorescence microscope; *M. tuberculosis *H37*Rv *and *M. bovis *were visualized with the optical microscope after Ziehl-Neelsen stain. To evaluate the adhesion of the bacteria to the cells, 20 fields for each slides were observed, and eventually it was calculated the main number of bacteria for each cell. To quantify the adhesion, also the standard deviation was calculated.

In order to evaluate the persistence of the bacteria inside the cells, it was evaluated the growth graph (point 0, 6h, 24h, 48h, 7 days). In particular, at each time of infection, cells were lysed with 1 ml of PBS/tryton 1% for 20 minutes and then 100 μl were plated in the solid medium 7H10 Middlebrook at 37°C for three weeks.

Supernatants obtained after 6, 24, 48 hours and 7 days were used to verify the expression of cytokines.

### Characterization of the immune response of the glial cells

In order to define the pattern of cytokines that characterize the immune response in the tubercular and paratubercular infection, it was used the kit Multi-Analyte ELISArray (Sabioscience) and the plates used have the following antibodies: ANTI IL-1A, IL-1B, IL-2, IL-4, IL-6, IL-10, IL-12, IL-17A, IFNγ, TNFα, G-CSF and GC-CSF.

Reading was performed at 450 nm, using the Microplate Elisa Reader (Molecular Devices). In the plate, standards for each cytokine searched were inserted in the plate and also negative controls, taken from non-infected cell cultures, in order to verify the background of cytokines produced by the cells. The value obtained from each cytokine subtracted from the negative control was compared with the reference table provided by the suppliers of the kit.

## Competing interests

The authors declare that they have no competing interests.

## Authors' contributions

SC is involved in the experimental design, performed infection experiment and elisa, and draft the manuscript. PM is involved in the experimental design and helped to draft the manuscript. AB participated in the design of the study. DU helped in the cell colture. EB provided the BV2 cell line. LAS and SZ conceived of the study, and participated in its design and coordination and helped to draft the manuscript. All authors read and approved the final manuscript.

Financial support: the study was funded by Grant FAR.
